# Screening for *Leishmania* spp. infection in patients treated with biologic agents for immune-mediated inflammatory diseases: results of an Italian multicentric prospective study

**DOI:** 10.1186/s41182-025-00802-9

**Published:** 2025-09-24

**Authors:** Emma Balducelli, Ana Torres, Sara Morselli, Andrea Angheben, Chiara Piubelli, Francesca Perandin, Salvatore Scarso, Cinzia Scambi, Alessandro Bartoloni, Lorenzo Zammarchi, Anna Barbiero, Filippo Lagi, Michele Spinicci, Francesca Nacci, Margherita Ortalli, Simone Baiocchi, Lorena Bernardo, Javier Moreno, Eugenia Carrillo, Stefania Varani

**Affiliations:** 1 Department of Medical and Surgical Sciences, University of Bologna, 40138 Bologna, Italy; 2 WHO Collaborating Centre for Leishmaniasis, Spanish National Center for Microbiology, Instituto de Salud Carlos III, Majadahonda, Spain; 3 Centro de Investigación Biomédica en Red de Enfermedades Infecciosas (CIBERINFEC), Instituto de Salud Carlos III, Madrid, Spain; 4 Department of Infectious, Tropical Diseases and Microbiology, IRCCS Sacro Cuore Don Calabria Hospital, 37024 Negrar di Valpolicella, Verona, Italy; 5 Rheumatology Unit, Section of Rheumatology Department of Internal Medicine, IRCCS Sacro Cuore Don Calabria Hospital, 37024 Negrar di Valpolicella, Verona, Italy; 6 Department of Clinical and Experimental Medicine, Università degli Studi di Firenze, 50134 Florence, Italy; 7 Infectious and Tropical Diseases Unit, Azienda Ospedaliero Universitaria Careggi, 50134 Florence, Italy; 8 Tuscany Regional Referral Center for Tropical Diseases, Azienda Ospedaliero Universitaria Careggi, 50134 Florence, Italy; 9 Section of Rheumatology, Department of Clinical and Experimental Medicine, Università degli Studi di Firenze, 50125 Florence, Italy; 10 Unit of Microbiology, IRCSS Azienda Ospedaliero-Universitaria di Bologna, 40138 Bologna, Italy

**Keywords:** *Leishmaniainfantum*, Asymptomatic *Leishmania* infection, Immune-mediated inflammatory diseases, Biologic agents

## Abstract

**Background:**

Escalation in the use of biologic agents including tumor necrosis factor (TNF)-α inhibitors to treat immune-mediated inflammatory diseases (IMID) is linked to higher susceptibility of severe infections caused by intracellular pathogens, including *Leishmania*.

**Methods:**

This multicentric prospective study assessed the presence of *Leishmania* spp. infection among patients with IMID under treatment with biologic agents in two Italian clinical centers. We utilized a combination of diagnostic tests: real-time PCR for the detection of parasitic kinetoplast DNA in peripheral blood, Western blot for the identification of serum IgG antibodies, and a Whole blood assay to assess cytokine and chemokine responses following stimulation with parasitic antigen.

**Results:**

A total of 126 patients residing in Italy were enrolled. Patients testing positive in at least one assay were classified as *Leishmania*-positive. Of the 125 asymptomatic individuals, 25 (20%) tested positive for *Leishmania* infection, revealing a significant rate of subclinical infection. The most frequent marker of infection was positive serology (15/126, 12%) followed by a detectable cell-mediated immune response (9/125, 7%). Parasitic DNA was detected in 3 patients (2%).

**Conclusions:**

This study showed a high prevalence of asymptomatic *Leishmania* infection in Italian patients with IMID under treatment with biologic agents, with a north-to-south gradient. Given the risk of disease reactivation, these patients may benefit from close monitoring. Further research is warranted to clarify the clinical implications of these findings.

## Background

*Leishmania* is an obligate intracellular protozoan parasite that primarily infects cells of the mononuclear phagocytic system and is transmitted by phlebotomine sand flies [1]. The clinical manifestations of leishmaniasis range from asymptomatic forms to localized cutaneous or mucosal infections and disseminated disease, which mainly involves the spleen, the liver and the bone marrow. The clinical presentation is influenced by the species and strain of the parasite, as well as by host-related factors, particularly the immune response [1, 2].

*Leishmania* parasites can persist for decades following treatment, as there appears to be no sterile immunity. Reactivation of symptomatic infection may occur when the host's immune system is impaired—for example, due to co-infection with HIV, immunosuppressive therapy following organ transplantation or the use of immunomodulatory drugs [2, 3].

Immunosuppressive drugs that can cause increased replication of the *Leishmania* parasite include corticosteroids, antimetabolites (such as Azathioprine or Methotrexate), calcineurine inhibitors (such as Cyclosporine), alkylating agents (such as Cyclophosphamide), TNF-α antagonists (such as Infliximab or Adalimumab), and certain monoclonal antibodies (such as Rituximab); chemotherapeutic agents used for hematologic malignancies can induce leishmaniasis reactivation as well [3]. Among all drugs, TNF-α inhibitors are particularly associated with increased risk.

Besides its role in human autoimmune diseases, TNF-α plays an important function in fighting intracellular pathogens, including *Mycobacterium tuberculosis*, *Listeria monocytogenes* and *Leishmania* spp. [4, 5]. The role of TNF-α in the control of leishmaniasis ranges from limiting the replication of the parasite up to eliciting an effective adaptative response; thus, TNF-α blockage can induce an increased replication of *Leishmania*, hence allowing the reactivation of the infection or more severe disease in case of newly acquired infections [4]. Most leishmaniasis cases occurring during treatment with TNF-α inhibitors were reported in the Mediterranean basin [5] and in Brazil [6], where *Leishmania infantum* circulates.

The aim of this study was to apply a combination of tests for the screening of *Leishmania* spp. infection in a cohort of patients living with IMID under treatment with TNF-α inhibitors or other biologics.

## Methods

### Study design

For this multicentric prospective study, patients with IMID under treatment with biologic agents were consecutively enrolled at the Department of Infectious, Tropical Diseases and Microbiology of Sacro Cuore - Don Calabria Hospital in Negrar, Verona (Northern Italy) and at the Infectious and Tropical Disease Unit of Careggi University Hospital in Florence (Central Italy). Inclusion criteria were age ≥ 18 years, at least 2 years of residence in Italy, treatment with biologic agents irrespective to the duration of treatment, and no past occurrence of visceral leishmaniasis (VL).

Blood samples were processed within 24 h from collection and tested for *Leishmania* spp. infection.

### Serological, molecular and immunological tests used to detect asymptomatic *Leishmania* infection

An asymptomatic *Leishmania* infection was defined as a condition in which an individual from an endemic region shows an immune response (either antibody-based or T-cell mediated) to the parasite, or has detectable *Leishmania* parasites or their DNA in the bloodstream, but does not exhibit any symptoms of illness [4]. Three different tests were used to screen for *Leishmania* spp. infection, including a Western blot to detect specific IgG against 14 kDa and 16 kDa leishmanial antigens (*Leishmania* WESTERN BLOT IgG kit (LDBio Diagnostics^®^, Lyon, France), a real-time Polymerase chain reaction (PCR) targeting the minicircles of kinetoplast (k) DNA as previously described [7] and a Whole blood assay (WBA), measuring the levels of two different cytokines (IL-2 and IP-10) by Cytokine bead array (CBA) in plasma from blood stimulated with soluble *L. infantum* antigens (SLA) as previously described [8]. In the latter, cytokine concentration (expressed in pg/mL) was calculated as the difference between SLA-stimulated and unstimulated (control) plasma concentrations, thereby reflecting the immune response specifically attributable to the presence of the *Leishmania *antigen.

### Statistical analysis

Statistical analyses were performed by using GraphPad Prism software (GraphPad Prism version 8.0.1 for Windows, GraphPad Software, San Diego, CA, USA). Categorical variables were analyzed using the Chi-square χ2 Fisher’s exact test, and statistical significance was determined at *p* < 0.05. The cut-off for IL-2 (6.56 pg/ml) and IP-10 (284.70 pg/ml) was determined by calculating the area under the receiver operating characteristic curve (AUC) and the 95% confidence intervals (CI) [0.87 (0.7828–0.9667) and 0.86 (0.7685–0.9576), respectively]. Patients exhibiting values above the cut-off for at least one cyto-chemokine were classified as WBA-positive, while patients with values below the cut-off were considered as WBA-negative. Normality was examined using the Shapiro–Wilk test. The Mann–Whitney U test was used to analyze differences between unpaired groups. Significance was set at *p* < 0.05.

To measure the concordance between different methods, the Kappa value was calculated. Results were interpreted according to the following Kappa values: (i) 0.01–0.20, slight agreement; (ii) 0.21–0.40, fair agreement; (iii) 0.41–0.60, moderate agreement; (iv) 0.61–0.80, substantial agreement; and (v) 0.81–1.00, perfect agreement. The numerical data used in the Figures and Tables are held in a public repository (10.6092/unibo/amsacta/8377).

## Results

### Patients’ characteristics

Between the 1st of September 2020 and the 30th of April 2021, 126 patients living with IMID were included in the study. The median age was 44 ± 14 years, patients were 61% male and 39% female. Of these, 74% were diagnosed with intestinal inflammatory disease, while 24% presented with rheumatological diseases and 2% with both types of disorders (Table 1). One hundred-thirteen (90%) out of 126 patients were under treatment with TNF-α inhibitors, while the remaining patients were treated with other biologics including Abatacept, Vedolizumab, Baricitinib and/or Tocilizumab (Table 1).

**Table 1:** Demographic and clinical characteristics of patients with immune-mediated inflammatory diseases (IMID) enrolled in the study (n = 126) and rates of *Leishmania* infection, as evaluated by Western blot (WB), real-time PCR and Whole blood assay (WBA)

Variables	Total positive n (%)	PCR positive	WB positive	WBA positive
Type of immune-mediated inflammatory diseases (IMID)				
Intestinal disorders (n = 93)	14/93 (15%)	1	7	6
Crohn’s disease (n = 51)	7/51 (14%)	0	4	3
Ulcerative colitis (n = 33)	5/33 (15%)	0	2	3
Ulcerative pancolitis (n = 9)	2/9 (22%)	1	1	0
Musculoskeletal disorders (n = 30)	11/30 (37%)	2	8	2
Seronegative arthritis (n = 3)	2/3 (67%)	0	2	0
Psoriatic arthritis (n = 3)	3/3 (100%)	1	3	0
Rheumatoid arthritis (n = 9)	1/9 (11%)	0	1	0
Spondyloarthritis (n = 15)	5/15 (33%)	1	2	2
Both (n = 3)	1/3 (33%)	0	0	1
Biological drugs				
Anti-TNF-α^a^ (n = 113)	23/113 (20%)	3	12	9
Other drugs^b^ (n = 13)	3/13 (23%)	0	3	0
**Sex**				
Female (n = 49)	8/49 (16%)	1	3	4
Male (n = 77)	18/77 (23%)	2	12	5
Patient’s residence (n = 126)				
Northern Italy (n = 109)	18/109 (17%)	1	10	9
Central and southern Italy (n = 13)	6/13 (46%)	2	3	2
Not known (n = 4)	2/4 (50%)	0	2	0

Anti-TNF-a drugs^a^: Adalimumab, Etanercept, Golimumab, Infliximab

Other drugs^b^ include: biotechnological drugs (Abatacept and Vedolizumab) as well as small molecules (Baricitinib and Tocilizumab)

Blood samples were analyzed for *Leishmania* spp. infection using three different techniques, and were considered positive if at least one of the methods yielded a positive result. Consequently, the control group was defined as those patients who tested negative in all three assays.

### Results of molecular, serological and immunological tests

Real-time PCR targeting *Leishmania* kDNA tested positive in 3 out of 126 patients (2%). In particular, one of these patients had high parasitemia levels (9 × 10^5^ equivalent parasites/mL) and exhibited suggestive symptoms of visceral leishmaniasis (VL), and was therefore diagnosed with overt disease. The two other PCR-positive patients had low parasite loads (6 × 10^–1^ and 2 × 10^–1^ equivalent parasites/mL, respectively) and were asymptomatic.

The presence of specific IgG antibody in serum was assessed using the WB, with 12% (15/126) of patients testing positive.

**Fig.1:**
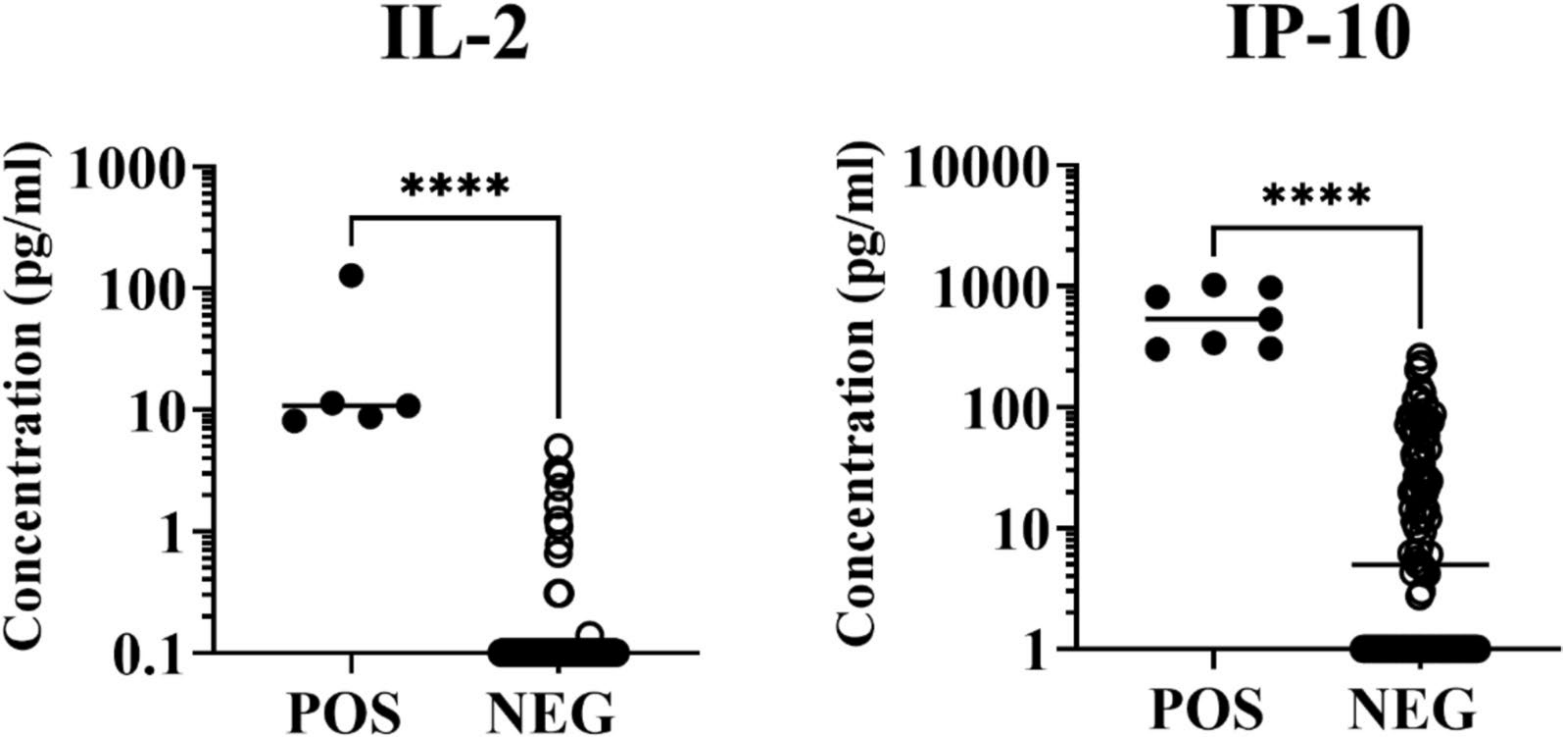
IL-2 and IP-10 levels in soluble leishmania antigen (SLA)-stimulated whole blood from patients with immune-mediated inflammatory diseases residing in a *L. infantum* endemic area. POS, positive; NEG, negative. Horizontal bars represent median concentrations. Each dot represents one subject. ****p < 0.0001 by using Mann–Whitney U test

WBA test was conducted in 125 out of the 126 patients. Nine patients (7.2%) tested positive to at least one of the WBA targets. Of these, 4 patients tested positive for IP-10 alone, 3 patients were positive for both IL-2 and IP-10, while 2 patients were positive for IL-2 alone. Patients with positive WBA showed significantly higher values for IL-2 (median 10.85 pg/mL; *p* < 0.001) and IP-10 (median 533.0 pg/mL; *p* < 0.010) when compared to WBA-negative individuals (median 0.1 pg/mL and 5 pg/mL respectively) (Fig. 1).

Among the 125 patients enrolled in the study who showed no symptoms of leishmaniasis, 25 were positive on at least one test, indicating a 20% prevalence of asymptomatic *Leishmania* infection in IMID. A significant difference emerged (*p* = 0.012) between the frequency of *Leishmania*-positive patients residing in northern Italy (17%, 18/109) and those residing in central and southern Italy (46%, 6/13). Concerning patients affected by intestinal IMID, 14% (7/51) of patients with Crohn's disease had asymptomatic *Leishmania* infection, compared with 17% (7/42) of patients with rectal ulcerative colitis or ulcerative pancolitis, showing no significant differences between baseline inflammatory bowel disease and the presence of *Leishmania* spp. infection.

Among patients with arthritis, differences were noticed in terms of frequency of positivity to *Leishmania* infection in patients with rheumatoid arthritis (1/9, 11%), seronegative arthritis (2/3, 67%), psoriatic arthritis (3/3, 100%), and spondylarthritis (5/15, 33%); however, the sample size was too small in each subgroup of patients to draw any conclusion. In addition, we did not detect any difference due to sex or age, among individuals who tested positive in any *Leishmania*-specific test and *Leishmania*-negative individuals.

### Concordance between tests in detecting *Leishmania* infection

**Fig.2:**
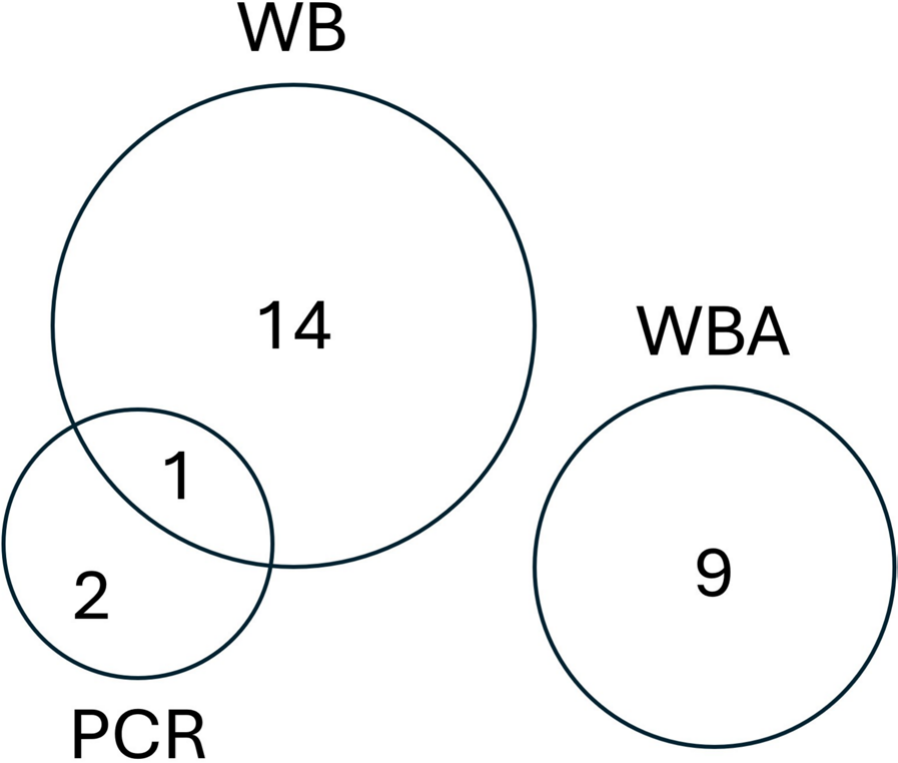
Venn diagram showing the agreement among PCR (Polymerase chain reaction), WBA (Whole blood assay) and WB (Western blot) in *Leishmania* positive patients (n = 26). The overlapping regions represent the concordance of positive results in two or more tests

Figure 2 presents a Venn diagram illustrating the overlap among PCR, WBA, and WB results in 26 patients who tested positive for at least one of the three methods; the VL case exhibited high parasitemia, tested positive by WB, but negative by WBA. Conversely, among patients with asymptomatic *Leishmania* infection (n = 25), none tested positive on at least two assays simultaneously. Contingency tables were used to assess concordance between the tests; each test showed poor or no agreement with the others (Cohen's kappa coefficients were − 0.278 for PCR/WBA, 0.293 for WB/WBA, and 0.074 for PCR/WB).

### Diagnostic accuracy of serological, molecular and immunological tests

When assessing the sensitivity of each test or test combination with respect to the definition of asymptomatic *Leishmania* infection (n = 25), we observed that WB exhibited the highest sensitivity as a single test (14 positive patients, 56%), while WBA had a sensitivity of 36% (9 positive patients) and PCR exhibited a sensitivity of 8% (2 positive patients, Table 2). The combination of WB and WBA provided the best performance, with a sensitivity of 92% (23 positive patients out of 25) for detecting asymptomatically infected individuals.

**Table 2:** Performance of single and combined tests in identifying asymptomatic *Leishmania* infection (n = 25)

	WB	WBA	PCR
Single test	Sens 56% NPV 90%	Sens 36% NPV 86%	Sens 8.0% NPV 81%
With PCR	Sens 64% NPV 92%	Sens 44% NPV 88%	–
With WBA	Sens 92% NPV 98%	–	–

*WB* Western blot, *WBA* Whole blood assay, *Sens* sensitivity, *NPV* negative predictive value

## Discussion

A number of evidence indicates that treatment with biologics, mainly TNF-α inhibitors predispose to the development of clinically evident leishmaniasis in patients with IMID [3, 5, 6].

To date, the European Crohn’s and Colitis Organization (ECCO) guidelines states that screening for parasitic infections should be considered in residents of endemic areas or with relevant travel history [9]. Further, whether leishmaniasis should be investigated in patients with IMID has been recently proposed in the research agenda by the European Alliance of Associations for Rheumatology [10]. Unfortunately, no gold standard test exists to detect asymptomatic *Leishmania* infection in this composite patient group [11].

Our results showed a high prevalence (20%) of asymptomatic *Leishmania* infection in Italian patients with IMID under treatment with biologic agents. By parasitic kDNA detection in blood, we identified a low frequency of asymptomatic *Leishmania* infection in patients with IMID (2% of total examined patients), in line with other studies in immunocompromised patients [7, 12]. We observed that most WBA-positive results were for IP-10, as 78% (7 out of 9) of the WBA-positive patients tested positive for this chemokine; this is consistent with recent findings in solid organ transplant recipients [7, 13] as well as in patients with IMID [8]. Additionally, WB showed the highest sensitivity as single test (56%). The combination of multiple tests increased the sensitivity in identifying this parasitic infection; WB plus WBA (including IP-10 quantification) proved to be the best test combination achieving a sensitivity of 92%, which is consistent with findings in transplant recipients from the same area [7]. Therefore, our data support the recommendation to use this combination to screen *Leishmania* spp. infection in patients living with IMID to align with the current recommendations [9]. In line with the previous study in transplant recipients [7], we observed that WB and WBA were positive on two separate groups of patients, with no individuals showing positivity to both tests. As a matter of fact, our finding suggests that a single screening tool would probably miss several *Leishmania* infection in patients living with IMID undergoing immunosuppression by biologic agents.

This study has several limitations, including the lack of follow-up for the tested patients as well as the small cohort size, as the heterogeneity of treatments and patient conditions may influence the results. Furthermore, no immunocompetent controls were included in the study to compare WBA findings. Additionally, other studies are needed in different epidemiological areas. On the other hand, the main strength of the study is its multicentric nature, with involvement of two clinical centers that were located in Northeastern and Central Italy, respectively. Consistent with previous findings in VL patients [14], we observed varying rates of asymptomatic *Leishmania* infection among IMID patients from different regions of Italy, with 17% *Leishmania*-positive patients in northern Italy and 46% in Central/Southern Italy, showing a north-to-south gradient based on the patient's residence.

## Conclusions

A high prevalence of asymptomatic *Leishmania* infection was observed in IMID patients treated with biologic agents, no association was found with the underlying disease. Further studies are needed to assess the reactivation rate of this parasitic infection in immunocompromised patients that are asymptomatically infected with *Leishmania.* Identifying immunocompromised patients carrying protozoan DNA in their blood could be valuable for controlling the spread of the parasite and preventing misdiagnosis, as VL often exhibits atypical presentation in the immunodeficient host.

## Data Availability

The datasets generated and analysed during the current study are available in the AMSActa Institutional research repository of the University of Bologna (10.6092/unibo/amsacta/8377).
